# Differential associations between retinal signs and CMBs by location: The AGES-Reykjavik Study

**DOI:** 10.1212/WNL.0000000000004792

**Published:** 2018-01-09

**Authors:** Chengxuan Qiu, Jie Ding, Sigurdur Sigurdsson, Diana E. Fisher, Qian Zhang, Gudny Eiriksdottir, Ronald Klein, Mark A. van Buchem, Vilmundur Gudnason, Mary Frances Cotch, Lenore J. Launer

**Affiliations:** From the Intramural Research Program (C.Q., J.D., Q.Z., L.J.L.), Laboratory of Epidemiology and Population Sciences, National Institute on Aging, NIH, Bethesda, MD; Aging Research Center (C.Q.), Department of Neurobiology, Care Sciences and Society, Karolinska Institutet–Stockholm University, Sweden; Icelandic Heart Association (S.S., G.E., V.G.), Kopavogur; Division of Epidemiology and Clinical Research (D.E.F., M.F.C.), National Eye Institute, NIH, Bethesda, MD; Ophthalmology and Visual Sciences (R.K.), University of Wisconsin Madison; Department of Radiology (M.A.v.B.), Leiden University Medical Centre, the Netherlands; and Faculty of Medicine (V.G.), University of Iceland, Reykjavik.

## Abstract

**Objective:**

To test the hypothesis that age-related macular degeneration (AMD) and retinal microvascular signs are differentially associated with lobar and deep cerebral microbleeds (CMBs).

**Methods:**

CMBs in lobar regions indicate cerebral amyloid angiopathy (CAA). β-Amyloid deposits are implicated in both CAA and AMD. Deep CMBs are associated with hypertension, a major risk factor for retinal microvascular damage. This population-based cohort study included 2,502 participants in the Age, Gene/Environment Susceptibility (AGES)-Reykjavik Study who undertook binocular digital retinal photographs at baseline (2002–2006) to assess retinal microvascular signs and AMD and brain MRI scan at both baseline and follow-up (2007–2011) to assess CMBs. We assessed retinal microvascular lesion burden by counting the 3 retinal microvascular signs (focal arteriolar narrowing, arteriovenous nicking, and retinopathy) concurrently present in the participant. We used multiple logistic models to examine the association of baseline retinal pathology to incident CMBs detected at follow-up.

**Results:**

During an average 5.2 years of follow-up, 461 people (18.3%) developed new CMBs, including 293 in exclusively lobar regions and 168 in deep regions. Pure geographic atrophy was significantly associated with strictly lobar CMBs (multivariable-adjusted odds ratio 2.59, 95% confidence interval [CI] 1.01–6.65) but not with deep CMBs. Concurrently having ≥2 retinal microvascular signs was associated with a 3-fold (95% CI 1.73–5.20) increased likelihood for deep CMBs but not exclusively lobar CMBs.

**Conclusions:**

Retinal microvascular signs and pure geographic atrophy may be associated with deep and exclusively lobar CMBs, respectively, in older people. These results have implications for further research to define the role of small vessel disease in cognitive impairment.

Cerebral microbleeds (CMBs) are a neuroimaging marker of cerebral small vessel disease (SVD) and are associated with stroke, cognitive decline, and dementia.^1–3^ Histopathologically, CMBs on T2*-weighted MRI correspond to small perivascular hemosiderin deposits.^1^ It has been assumed that CMBs in the deep or infratentorial areas reflect hypertensive microangiopathy, whereas lobar CMBs represent a marker for cerebral amyloid angiopathy (CAA).^1,4^

Microvasculature in the retina and the brain shares embryologic origin, anatomic features, metabolic activities, and similar patterns of vascularization and extracellular deposits.^5^ Retinal microvascular signs (e.g., focal arteriolar narrowing and arteriovenous nicking) are thought to result partly from short- or long-term exposures to cardiovascular risk factors, especially hypertension.^6^ Population-based cohort studies have related retinal microvascular signs to white matter lesions (WMLs), lacunar infarcts, and subcortical infarcts.^7–9^ However, the longitudinal association of retinal microvascular pathology with CMBs by location has yet to be examined.

Age-related macular degeneration (AMD) is a common retinal degenerative disorder among older people. Evidence has emerged that β-amyloid deposits, a hallmark of Alzheimer disease (AD), may be involved in the pathogenesis of AMD.^10,11^ Thus, retinal amyloid plaque has been proposed as a potential biomarker for AD and amyloid deposits in the brain.^12–14^

The cross-sectional data from the population-based Age, Gene/Environment Susceptibility (AGES)-Reykjavik Study showed an association of CMBs with retinal microvascular signs^15^ and a suggestive association with pure geographic atrophy.^16^ In this follow-up study, we seek to investigate the longitudinal associations of retinal microvascular signs and AMD with CMBs at specific locations in the brain. We hypothesize that the load of retinal microvascular signs is associated specifically with deep CMBs due primarily to hypertensive microangiopathy, whereas AMD is associated with lobar CMBs due predominantly to CAA.

## Methods

### Study population

This is a longitudinal population-based cohort study. Participants were from the AGES-Reykjavik Study that aimed at investigating genetic and environmental factors contributing to clinical and subclinical diseases in aging, as fully described elsewhere.^17^ Briefly, the Reykjavik Study was launched in 1967 by the Icelandic Heart Association and included a cohort of men and women born from 1907 to 1935. In 2002 to 2006, survivors of the cohort were invited to participate in the AGES-Reykjavik Study, and 5,764 persons were examined (baseline, AGES-I). In 2007 to 2011, survivors of the AGES-I cohort were invited for follow-up examination, and 3,316 were reassessed (follow-up, AGES-II). In total, 2,672 persons underwent brain MRI scans in both AGES-I and AGES-II and had the brain images needed for assessment of CMBs.^18^ Of these, we excluded 133 persons who had no data of either AMD or retinal vascular signs and additional 37 individuals who were diagnosed with prevalent dementia at baseline, leaving 2,502 persons for the present analysis. Of these, data on AMD and retinal microvascular signs were available in 2,484 and 2,497 persons, respectively.

### Standard protocol approvals, registrations, and patient consents

The AGES-Reykjavik Study was approved by the Icelandic National Bioethics Committee (VSN-00-063), which acts as the Institutional Review Board of the Icelandic Heart Association, and by the Institutional Review Board for the National Institute on Aging, NIH. Written informed consent was obtained from all participants.

### Fundus photography and assessments

Digital fundus photographs were taken at baseline (2002–2006) following standardized protocols.^19^ Briefly, after the maximal pharmacologic dilation of the pupils, two 45° retinal images, centered on the optic disc and the fovea, were taken of each eye with a 6.3-megapixel Canon digital nonmydriatic camera (Canon, Lake Success, NY). EyeQ Lite software (an image-processing database for storage, retrieval, and manipulation of digital retinal images) was used for retinal image acquisition, assessment, and archiving.

Using the modified Wisconsin Age-Related Maculopathy Grading scheme, graders masked to participants' identity evaluated digital retinal images at the University of Wisconsin Ocular Epidemiology Reading Center for AMD lesions.^19^ Early AMD was defined by the presence of any soft drusen and pigmentary abnormalities or the presence of large soft drusen. Late AMD was defined by the presence of either geographic atrophy or signs of exudative AMD (i.e., subretinal hemorrhage, subretinal fibrous scar, retinal pigment epithelial detachment, sensory retinal detachment, and any signs of treatment for neovascular AMD). Reassessments on randomly selected images of 25 eyes showed excellent intraobserver (κ = 0.82–1.0) and interobserver (κ = 0.88–1.0) agreement on AMD classification.^19^

Retinal microvascular signs were assessed following a validated protocol.^20^ Retinal focal arteriolar narrowing or arteriovenous nicking was defined as present if the grader was at least 90% certain that the lesion was present in a given eye. Retinopathy lesions, classified similarly, included retinal blot hemorrhages, microaneurysms, soft exudates, and more advanced lesions associated with diabetic retinopathy. The intraobserver and interobserver agreement was fair to good (κ = 0.31–0.76) for focal arteriolar signs and excellent (κ = 0.81–1.00) for retinopathy lesions.^21^

### MRI acquisition and reading protocol

All eligible participants had high-resolution brain MRI acquired on a 1.5T Signa TwinSpeed System (General Electric Medical Systems, Waukesha, WI) at both the AGES-I and AGES-II examinations.^18,22^ Briefly, the core protocol included a T1-weighted 3-dimensional spoiled gradient-echo sequence, a proton density/T2-weighted fast spin-echo sequence, a fluid-attenuated inversion recovery sequence, and a T2*-weighted gradient-recalled echo sequence. MRI scan parameters for all sequences are fully reported elsewhere.^15,22^ All images were acquired to give full brain coverage, and slices were angled parallel to the anterior commissure-posterior commissure line to give reproducible image views in the oblique-axial plane.

All brain images were analyzed automatically with the AGES/Montreal Neurological Institute image postprocessing pipeline,^22^ which segments the whole brain into gray matter, normal white matter, WMLs, and CSF. Brain infarcts were defined as defects in the brain parenchyma with a signal intensity that was isointense to that of CSF on all sequences with a diameter ≥4 mm, except for infarcts in the cerebellum, brainstem, and cortex, which had no size criteria.^21^

### Assessment and categorization of CMBs

CMBs were defined as a focal area of signal void within the brain parenchyma that is visible on the T2*-weighted gradient-recalled echo images and smaller or invisible on T2-weighted images. The semiquantitative assessment of CMBs is conducted at a computer workstation with customized in-house software for the longitudinal image analysis. To maximize comparability of baseline and follow-up images, they were aligned with each other with a linear coregistration and viewed side by side on the computer screen. This involved registering both the baseline and follow-up proton density/T2-weighted, T2*-weighted, and fluid-attenuated inversion recovery images for each participant to the baseline T1-weighted 3-dimensional spoiled gradient-echo images because the T1s had the highest spatial resolution of all images in the protocol. By performing this coregistration, we removed differences in image slice alignments due to different positioning of the participant in the head coil and different positioning of slices by the operator of the MRI scanner at the time of scanning in the 2 visits. The follow-up MRI scans were read by investigators blinded to the baseline scans. If a CMB was found on the follow-up MRI scan, the baseline MRI scan was examined to determine whether the CMB was present in the same location of the slice. If so, the follow-up CMB was labeled prevalent; if not, the CMB was labeled incident. This image preprocessing and rating procedure made it possible to reliably distinguish incident CMBs from prevalent CMBs. Both the intrarater agreement and interrater agreement based on 2 ratings within a 6-month interval were good (κ range 0.70–0.75).^18^

CMB location was recorded for lobar (frontal, temporal, parietal, and occipital) and deep or infratentorial (basal ganglia, thalamus, corpus callosum, brainstem, and cerebellum) regions. Because we were interested in CMBs with an amyloid (CAA) or vascular (hypertensive microangiopathy) origin according to our hypothesis and because it is possible that lobar CMBs are also related to hypertension, we classified people with CMBs by location as follows: people with at least 1 new CMB restricted to lobar regions were considered to have lobar CMBs exclusively, and those with CMBs in a deep or infratentorial region with or without concomitant lobar CMBs were classified as having deep CMBs.^2,18^

### Potential confounders

Demographics, cardiovascular risk factors, ischemic cerebral SVD, and *APOE* genotype at baseline may confound the association between retinal signs and incident CMBs. Age, sex, education, and smoking were assessed via questionnaire administered by a trained interviewer.^17^ Body mass index (BMI) was calculated from measured weight (kilograms) divided by height (meters) squared. Fasting blood glucose and total cholesterol were measured according to standard protocols. Use of medications (e.g., antihypertensive and hypoglycemic agents) was ascertained from medication vials brought to the study center. Arterial blood pressure was measured twice in a sitting position to the nearest 2 mm Hg with a mercury sphygmomanometer with a standard-sized cuff. Hypertension was defined as having a self-reported history of hypertension, having a blood pressure ≥140/90 mm Hg, or using antihypertensive drugs. Diabetes mellitus was defined according to self-reported history of diabetes mellitus, use of hypoglycemic drugs, insulin injection, or fasting blood glucose level ≥7.0 mmol/L.^15^ *APOE* genotype was determined with standard DNA amplification and restriction isotyping.^23^

### Statistical analysis

Baseline characteristics of participants were presented by incident CMBs detected at follow-up. General linear or logistic regression analysis was performed to compare means or proportions with adjustment for age and sex. To assess the extensity and severity of retinal vascular lesions, we created an index for retinal microvascular burden by counting the 3 retinal microvascular signs concurrently present in the participant (range 0–3), i.e., retinal focal arteriolar narrowing, arteriovenous nicking, and retinopathy lesions.^24^ The index was categorized into 0 (reference), 1, and ≥2; we combined the index scores of 2 and 3 because of the low number of participants who had a score of 3 (n = 9). We used binomial logistic models to estimate the odds ratio and 95% confidence interval of incident CMBs associated with AMD and the index of retinal microvascular signs. Multinomial logistic regression models were constructed to examine the association between retinal signs and CMBs by location (deep vs strictly lobar areas).^2,18^ We reported the main results from the model that was adjusted for age, sex, types of coil, follow-up interval, current smoking, BMI, systolic blood pressure, use of antihypertensive medications, total cholesterol, diabetes mellitus, MRI markers of cerebral SVD (i.e., subcortical infarcts, volume of WMLs, and prevalent CMBs) assessed at baseline, and *APOE* ε4 allele. Stata Release 12 (StataCorp 2011, StataCorp LP, College Station, TX) was used for all analyses.

## Results

### Baseline characteristics of participants

At baseline, the mean age of the 2,502 participants was 74.6 (SD = 4.7) years, and 58.63% were women. After an average 5.2 (SD = 0.2) years of follow-up, new CMBs were detected in 461 (18.43%) persons; of these, 293 (63.56%) had CMBs exclusively in lobar regions and 168 (36.44%) in deep brain regions. Compared with persons who did not develop new CMBs at follow-up, those who developed incident CMBs were older; were more likely to be male, smoke, and have diabetes mellitus; and had a lower BMI, a higher level of diastolic blood pressure, and a larger volume of WMLs, subcortical infarcts, and prevalent CMBs at baseline (all *p* < 0.05), but the 2 groups did not differ significantly in educational attainments, systolic blood pressure, hypertension, use of antihypertensive drugs, total cholesterol, and *APOE* ε4 allele (table 1).

**Table 1:**
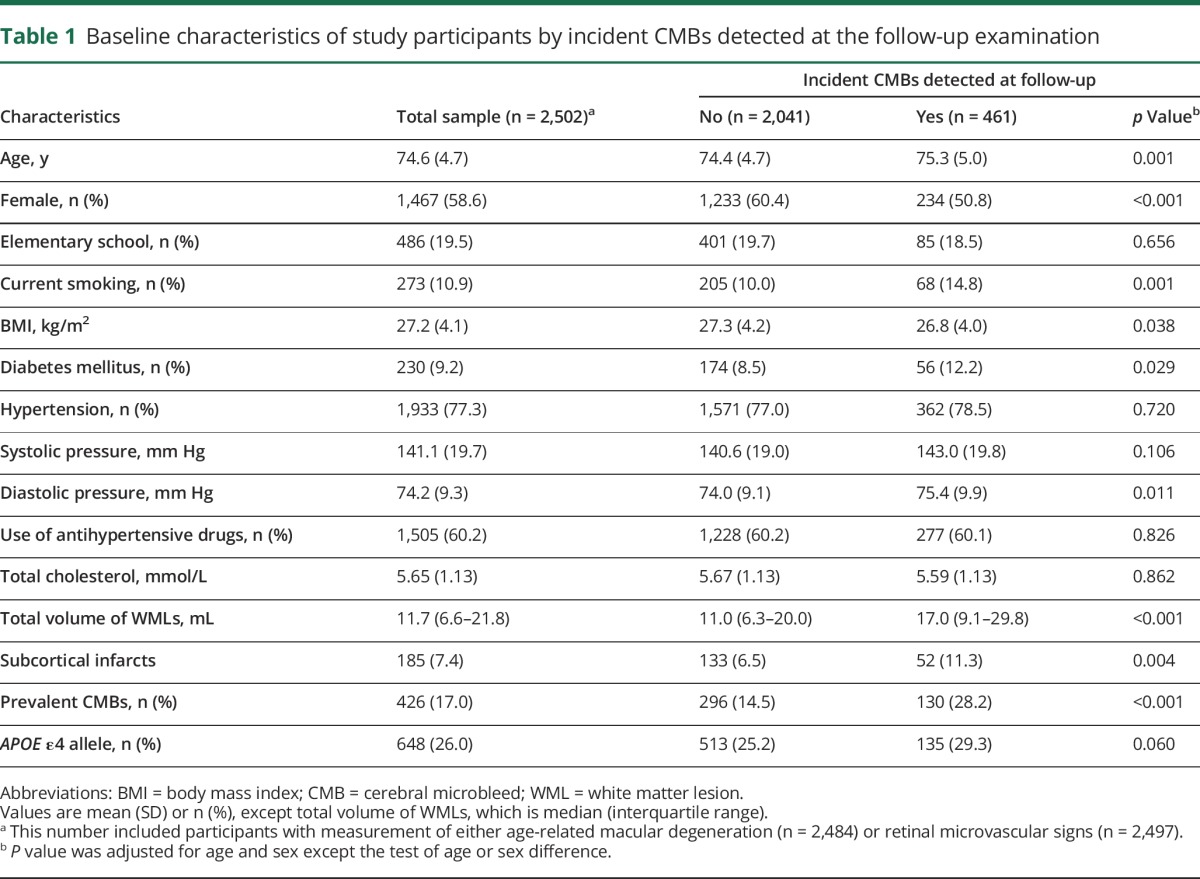
Baseline characteristics of study participants by incident CMBs detected at the follow-up examination

### AMD subtypes and CMBs by anatomic location

At baseline, of the 2,484 persons, early AMD was detected in 451 (18.16%) and late AMD in 92 (3.70%); 63 (2.54%) of those with late AMD had exudative AMD, and 29 (1.12%) had pure geographic atrophy. After adjustment for multiple potential confounders, including baseline MRI markers of cerebral SVD (e.g., prevalent CMBs, subcortical infarcts, and volume of WMLs), early AMD was not significantly associated with global or regional incident CMBs (table 2). However, pure geographic atrophy, but not exudative AMD, was significantly associated with an ≈2.5-fold increased likelihood of developing new CMBs exclusively in the lobar areas. Neither exudative AMD nor pure geographic atrophy was significantly associated with deep CMBs (table 2).

**Table 2:**
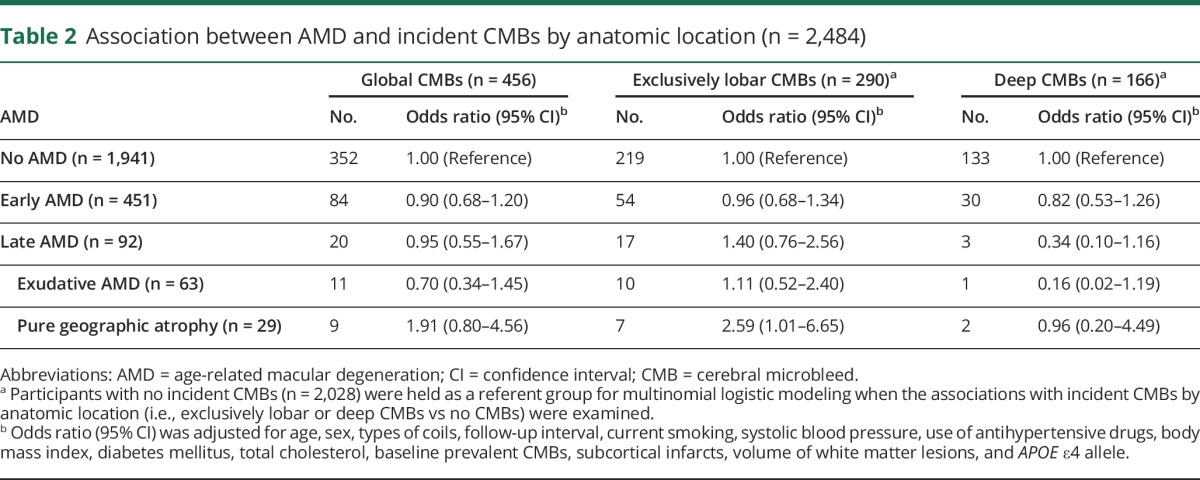
Association between AMD and incident CMBs by anatomic location (n = 2,484)

### Retinal microvascular signs and CMBs by anatomic location

After controlling for multiple potential confounders, including baseline imaging markers of cerebral SVD, having retinal focal arteriolar narrowing and arteriovenous nicking was significantly associated with an increased likelihood of developing new CMBs specifically in the deep brain areas, not exclusively in the lobar regions (table 3). The presence of retinopathy lesions was significantly associated with an increased likelihood of having global CMBs. When the 3 retinal microvascular signs were aggregated into a composite index, we found that, compared to persons having none of the 3 retinal microvascular signs, each additional lesion was associated with a significantly increased likelihood of developing new CMBs globally (*p* for trend = 0.047), especially in deep brain regions (*p* for trend <0.001); concurrently possessing ≥2 retinal microvascular signs was significantly associated with a 3-fold increased likelihood of developing deep CMBs, whereas there was no significant association between the index of retinal microvascular signs and the likelihood of having strictly lobar CMBs (table 3).

**Table 3:**
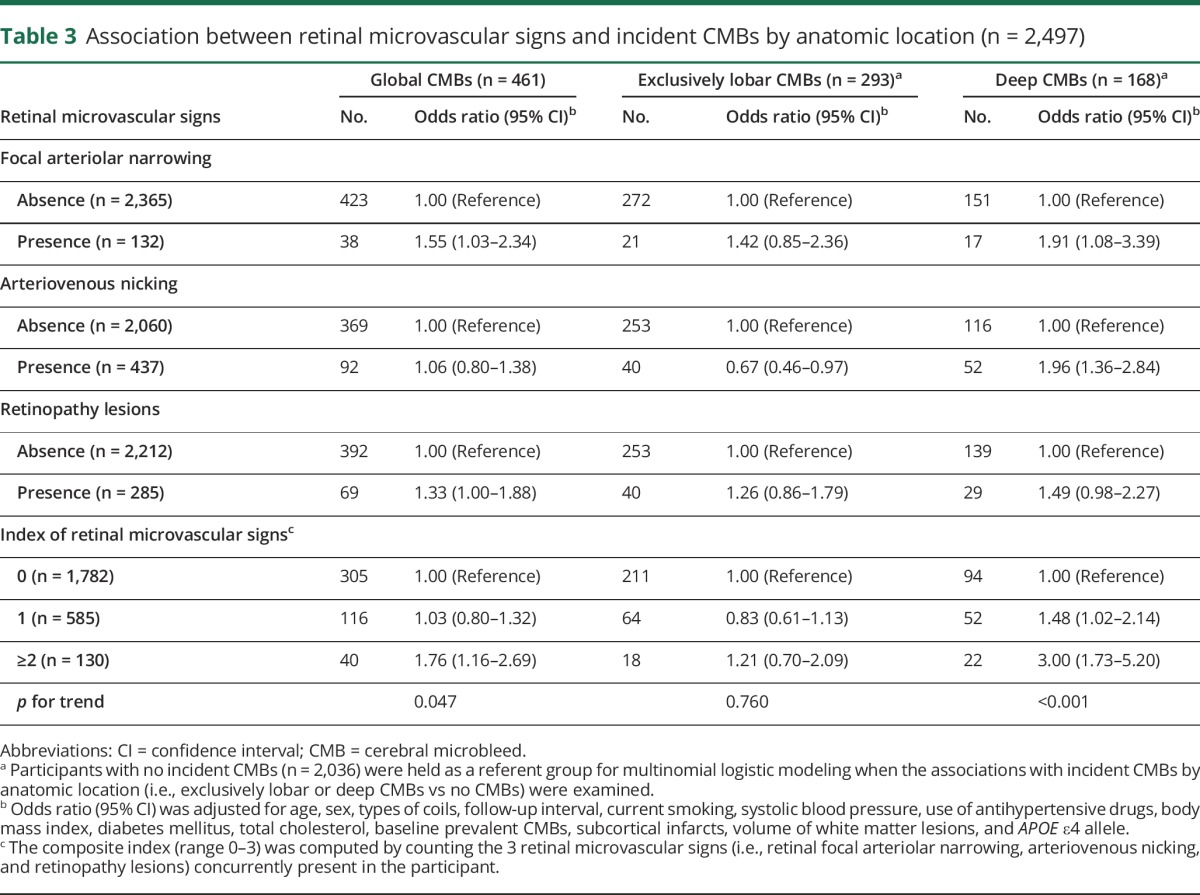
Association between retinal microvascular signs and incident CMBs by anatomic location (n = 2,497)

### Additional analyses

First, we repeated the analyses by excluding persons with prevalent CMBs at baseline (n = 427), which yielded results similar to those reported in tables 2 and 3 (table e-1, http://links.lww.com/WNL/A18). Furthermore, there was no significant association between AMD and exclusively deep CMBs, and the associations of retinal microvascular signs (individual signs or the composite index) with exclusively deep CMBs were less evident compared to those associations with the presence of any deep CMBs (data not shown). Finally, there was no statistical interaction of *APOE* ε4 allele with either retinal vascular signs or AMD for incident CMBs, and analysis stratified by *APOE* ε4 did not show any additional significant associations between retinal signs and incident CMBs (data not shown).

## Discussion

In a well-established cohort of older adults, we found evidence suggesting that pure geographic atrophy might be associated with an increased risk of developing CMBs exclusively in lobar areas but not in deep brain regions. Early AMD and exudative AMD were not associated with developing CMBs. In contrast, a greater number of retinal microvascular signs was associated with a higher likelihood of developing new CMBs in deep brain regions and not with CMBs only in lobar areas. The observed associations are present even after controlling for demographics, cardiovascular risk factors, *APOE* ε4 allele, and markers of cerebral SVD (e.g., prevalent CMBs, subcortical infarcts, and WMLs). Given the underlying presumed etiopathology of CMBs by location, this suggests that pure geographic atrophy and load of retinal microvascular lesions may be useful markers of different underlying pathologies in the brain.

The association between AMD and an increased risk of clinical stroke has been reported in several population-based cohort studies.^25,26^ In this cohort study, we examined the longitudinal associations of retinal microvascular signs and macular degeneration with CMBs by anatomic location. Our findings suggest a new hypothesis that pure geographic atrophy subtype of AMD may be associated with processes also regulating cerebral neurodegeneration, insofar as CAA, which is caused by amyloid deposits in the lobar microvessels, does.^1,4^ It is worth noting that the prevalence of pure geographic atrophy in our sample was only 1.12%, so results should be interpreted with caution.

However, the far more prevalent hypertension is the major risk factor for retinal arteriolar signs and retinopathy lesions, as well as for cerebrovascular disease ranging from clinical stroke and subclinical brain lesions (e.g., lacunar infarcts, WMLs, and CMBs) to progression of cerebral SVD.^9,27–29^ Our study showed that the more retinal microvascular signs present, the higher the likelihood of developing new CMBs in deep brain areas, not in strictly lobar regions. This pattern of association is in line with a hospital-based study^30^ that showed that retinal focal arteriolar narrowing and arteriovenous nicking were less common in patients with lobar intracerebral hemorrhage than in those with deep intracerebral hemorrhage, a sequelae of CMBs.^1,2^

The pathophysiologic mechanisms underlying the associations of retinal microvascular signs and geographic atrophy with region-specific CMBs are not fully understood, but studies of the etiopathophysiology of this association are emerging. First, retinal arteriolar signs and deep CMBs share common cardiovascular risk factors, especially hypertension.^6^ Second, disruption of the blood-retina barrier from retina hypoxia owing to long-term exposures to cardiovascular risk factors, which is analogous to and correlated with the blood-brain barrier dysfunction, may play a part in the pathogenesis of both retinal microvascular lesions and cerebral microangiopathy.^30,31^ Finally, evidence has emerged that β-amyloid, as a hallmark of Alzheimer pathology and CAA in the brain,^32,33^ also exists in the retina and the heart of patients with AD.^34–36^ Given the homology of microvasculature in the brain and the retina and the involvement of β-amyloid in both retinal degeneration (AMD) and CAA,^10,12,37,38^ the link of pure geographic atrophy with strictly lobar CMBs may reflect underlying pathologic processes common in both the brain and the retina. Given that experimental and clinical studies have shown amyloid deposits in the retina, the hypothesis of neurodegenerative-like process in AMD is of interest to follow up in populations in which the prevalence of pure geographic atrophy or, in reverse, AD is higher than in our cohort.

Strengths of this study include the prospective nature of the study design, the community-derived large cohort of older adults, and control of multiple potential confounders, including markers of cerebral SVD. Furthermore, the MRI sequences and definition of CMBs used in our study were comparable to those in the recommended standard scales (e.g., Microbleed Anatomical Rating Scale [MARS] and Brain Observer MicroBleed Scale [BOMBS]).^1^ Our study also has limitations. First, the analytic sample was younger and healthier than those excluded, which may potentially bias our estimates. Second, a potential limitation of the retinal microvascular index was that the 3 retinal microvascular signs might reflect different stages of retinal microvascular alterations. However, the composite index of retinal microvascular profile may be more stable than the individual retinal makers because single retinal microvascular signs may fluctuate as a result of transient metabolic and hemodynamic alterations.^39^ Finally, statistical power was limited for the analysis of certain subgroups (e.g., deep CMBs in late AMD).

Overall, our findings suggest that additional studies are necessary to explore common pathophysiologic processes of microvascular damage and neurodegeneration in the retina and the brain. In combination with additional biological and functional information, it may be possible in the future to develop retinal screening algorithms for brain disease and to identify elderly people at high risk for CMBs and related functional consequences.

## Glossary

AD: Alzheimer disease
AGES: Age, Gene/Environment Susceptibility
AMD: age-related macular degeneration
BMI: body mass index
CAA: cerebral amyloid angiopathy
CMB: cerebral microbleed
SVD: small vessel disease
WML: white matter lesion
